# Blastomere movement post first cell division correlates with embryonic compaction and subsequent blastocyst formation

**DOI:** 10.1186/s12958-019-0488-5

**Published:** 2019-05-15

**Authors:** Kazuki Ohata, Kenji Ezoe, Tetsuya Miki, Hirofumi Morita, Ryoma Tsuchiya, Shigeru Kaneko, Tadashi Okimura, Kazuo Uchiyama, Akiko Yabuuchi, Tamotsu Kobayashi, Markus Montag, Keiichi Kato

**Affiliations:** 1 Kato Ladies Clinic, 7-20-3 Nishishinjuku, Shinjuku-ku, Tokyo, 160-0023 Japan; 2 ilabcomm GmbH, Eisenachstr, 34, 53757 Sankt Augustin, Germany

**Keywords:** Blastocyst, Blastomere movement, Compaction, First cell division, Time-lapse system, Live birth

## Abstract

**Background:**

Blastomere movement (BMov) occurs after the first cell division in human embryos. This movement has been suggested as a prognostic parameter for pregnancy outcome prediction following cleavage-stage embryo transfer. However, the effect of BMov on preimplantation development and pregnancy outcome after blastocyst transfer remains unclear. Therefore, this study aimed to evaluate whether BMov after the first cell division is correlated with blastocyst formation rate and live birth rate after single vitrified-warmed blastocyst transfer (SVBT).

**Methods:**

Nine hundred and sixty-six embryos cultured in the EmbryoScope+® time-lapse system were retrospectively analyzed. The BMov type was categorized into three groups; namely, bouncing, wobbling, and twist-and-crumble. The BMov duration (dBMov) between the first (t2) and second cell division (t3) was monitored, and the ratio of dBMov to the duration of the 2-cell stage was calculated [dBMov/(t3-t2)]. Developmental rates to the 4-cell, 8-cell, morula, blastocyst, and expanded blastocyst stages were assessed, as well as blastocyst morphological grade. The correlations between dBMov and clinical pregnancy, ongoing pregnancy, and live birth rates were evaluated.

**Results:**

Increased dBMov/(t3-t2) was significantly correlated with decreased developmental rates to the 8-cell, morula, blastocyst, and expanded blastocyst stages, especially from the 4-cell stage to the morula stage. Analysis of different types of BMov revealed that embryos with bouncing movement exhibited significantly higher developmental rates to the 8-cell, morula, blastocyst, and expanded blastocyst stages compared with embryos with twist-and-crumble movement. The morphological quality of blastocyst-stage embryos with twist-and-crumble movement was significantly lower than that of embryos with bouncing and wobbling movements. The rates of clinical pregnancy, ongoing pregnancy, and live birth after SVBT were not correlated with BMov type or duration.

**Conclusions:**

Embryonic compaction and subsequent blastocyst formation are adversely affected by twist-and-crumble movement and prolonged movement after the first cell division. Our results indicate that the preimplantation developmental competence of human embryos could be predicted by assessing BMov after the first cell division on day 1.

**Electronic supplementary material:**

The online version of this article (10.1186/s12958-019-0488-5) contains supplementary material, which is available to authorized users.

## Introduction

The development of time-lapse imaging technology has enabled consecutive observation of fertilization events, embryonic cleavage, compaction, and blastulation under stable and uninterrupted conditions, thus allowing the selection of appropriate embryos for transfer [1–7]. Several studies that have utilized time-lapse systems have reported that blastomere movement (BMov) occurs after the first cell division in human embryos [8, 9]. A recent study reported that BMov type (bouncing, wobbling, or twist-and-crumble) and duration vary markedly among embryos [10]. Furthermore, this study showed BMov duration to be correlated with the incidence of asymmetric division, fragment generation, and ongoing pregnancy rate after fresh cleaved-embryo transfer on day 2. Therefore, BMov is considered a prognostic parameter for outcome prediction following cleavage-stage embryo transfer. However, it is not known whether BMov correlates with blastocyst formation and pregnancy outcome after blastocyst transfer. We evaluated the association between the BMov type and duration (dBMov) post first cell division and preimplantation development and live birth rates (LBRs) after blastocyst transfer.

## Materials and methods

### Study population and design

We retrospectively analyzed the clinical records of 966 cleaved embryos from 634 treatment cycles of 634 women who underwent oocyte retrieval during clomiphene citrate-based minimal-stimulation cycle, and which were scheduled for planned freeze-all strategy at the blastocyst stage at Kato Ladies Clinic between April 2017 and May 2018. Oocytes were inseminated by intracytoplasmic sperm injection (ICSI). The patients’ own oocytes were used during treatment. The patients presenting with recurrent implantations; i.e., those who had previously undergone embryo transfer four or more times, were excluded from the study [11]. In addition, patients who underwent preimplantation genetic diagnosis and women with hypothalamus-pituitary gland-related amenorrhea were excluded. Embryos that were directly cleaved from the 1- to 3-cell stage at the first cleavage were excluded from annotation.

### Analysis of Blastomere movement

The detailed protocol for minimal stimulation with clomiphene citrate, ICSI, and embryo culture has been previously reported [10, 12]. The time points at which the second (t2) and third cells (t3) were completely separated by confluent membranes was annotated using the EmbryoScope+® time-lapse system, according to a previous report [6]. The type of BMov after the first cell division was monitored, as previously reported [10]. The dBMov between the first (t2) and second cell divisions (t3) was annotated, and the ratio of dBMov to the duration of the 2-cell stage (t3-t2) was calculated as dBMov/(t3-t2). The type of BMov was categorized into blastomere bouncing, wobbling, and twist-and-crumble groups, as previously reported [10]. In brief, when blastomeres temporally shrank and then expanded immediately after the first cell division, blastomere movement was categorized as “bouncing”. Cytoplasmic and membrane waving/distortion, which was reflected by a continuous change in the blastomere shape after t2, was defined as blastomere “wobbling”. Blastomere rolling followed by fragment generation was defined as “twist-and-crumble”. All annotations in the Embryo Viewer software and conventional morphological grading of embryos were performed by four blinded operators with more than 10 years of experience in embryology, who were unaware of the clinical outcomes.

### Blastocyst transfer

Single vitrified-warmed blastocyst transfers (SVBTs) were performed, as previously reported [12]. Dydrogesterone (30 mg/d) was administered orally during the early luteal phase after blastocyst transfer. In cases with insufficient luteal function, progesterone was administered intramuscularly (125 mg/d) or intravaginally (300–800 mg/d) until the 9th week of pregnancy. The clinical-pregnancy and ongoing-pregnancy rate (CPR and OPR, respectively) and LBR were analyzed.

### Statistical analysis

All statistical analyses were performed using JMP software (SAS Institute, Cary, NC, USA). Proportion data were analyzed using the chi-square test or Cochran-Armitage test. Continuous parameters were compared via one-way analysis of variance (ANOVA), with significance determined using Tukey’s test for post-hoc analysis. Logistic regression was used to assess the contributing strength of parameters that are potentially associated with pregnancy outcome. Spearman’s Rank Correlation Coefficient (SRCC) was used to measure the degree of association between two continuous variables. Adjusted odds ratios were reported with 95% confidence intervals for each group. A value of *P* <  0.05 was considered statistically significant.

## Results

Patient characteristics are shown in Table 1. The characteristics and clinical outcomes of the embryos are shown in Table 2. The proportions of embryos with bouncing, wobbling, and twist-and-crumble movements were 56.2, 20.1, and 23.7%, respectively. Mean dBMov was 3.84 ± 0.07 h, and the value of dBMov/(t3-t2) was 0.366 ± 0.007. The type of blastomere movement was not correlated with age of the female (bouncing, 39.1 ± 0.2; wobbling, 39.2 ± 0.2; and twist-and-crumble, 38.8 ± 0.2, *P* = 0.4746) or the male (bouncing, 41.0 ± 0.2; wobbling, 41.5 ± 0.4; and twist-and-crumble, 41.0 ± 0.4, *P* = 0.2547). Furthermore, dBMov was not associated with the patient’s age (SRCC: female age, 0.0135, *P* = 0.6820; male age, 0.0551, *P* = 0.0942) and dBMov ratio (SRCC: female age, 0.030, *P* = 0.3516; male age, 0.0576, *P* = 0.0743) were not associated with the patient’s age.

**Table 1:** Patient characteristics (*n* = 634)

Age (years)	
Female	39.2 ± 0.1 [27–47]
Male	41.1 ± 0.2 [25–64]
No. of previous embryo transfer cycles	1.62 ± 0.03 [0–3]
BMI	21.2 ± 0.1 [15.7–33.7]
Cause of infertility	
Ovulation	7 (1.1)
Tubal factor	15 (2.4)
Endometrial factor	45 (7.1)
Male factor	110 (17.4)
Combined	29 (4.6)
Unexplained	428 (67.5)

Continuous data are presented as mean ± standard error of the mean [range], categorical data are presented as n (%). Abbreviations: *BMI* body mass index

**Table 2:** Analyzed embryo characteristics and clinical outcomes

		Blastomere movement type		
	Total	Bouncing	Wobbling	Twist-and-crumble
No. of cleaved embryos analyzed	966	543 (56.2)	194 (20.1)	229 (23.7)
Four-cell stage embryos	964 (99.8)	543 (100)	194 (100)	228 (99.6)
Eight-cell stage embryos	900 (93.2)	516 (95.0)^a^	181 (93.3)^a^	202 (88.2)^b^
Morula	842 (87.2)	493 (90.8)^a^	167 (86.1)^a,b^	180 (78.6)^b^
Blastocysts	731 (75.7)	435 (80.1)^a^	144 (74.2)^a,b^	152 (66.4)^b^
Blastocysts expanded	681 (70.5)	406 (74.8)^a^	136 (70.1)^a,b^	139 (60.7)^b^
Blastocysts cryopreserved	625 (64.7)	378 (69.6)^a^	124 (63.9)^a^	123 (53.7)^b^
Morphological grade of blastocysts				
ICM				
Grade A	291 (46.6)	184 (48.7)	59 (47.6)	48 (39.0)
Grade B	214 (34.2)	129 (34.1)	37 (29.8)	48 (39.0)
Grade C	120 (19.2)	65 (17.2)	28 (22.6)	27 (22.0)
TE				
Grade A	267 (42.7)	177 (46.8)^a^	56 (45.2)^a^	34 (27.6)^b^
Grade B	178 (28.5)	97 (25.7)^a^	35 (28.2)^a,b^	46 (37.4)^b^
Grade C	180 (28.8)	104 (27.5)	33 (26.6)	43 (35.0)
No. of blastocysts thawed	499	298	102	99
No. of survived blastocysts	496 (99.3)	297 (99.7)	101 (99.0)	98 (99.0)
No. of blastocysts transferred	496	297	101	98
Morphological grade of blastocysts transferred				
ICM				
Grade A	250 (50.3)	157 (52.7)	52 (51.5)	41 (41.8)
Grade B	158 (31.9)	90 (30.4)	33 (32.7)	35 (35.7)
Grade C	88 (17.8)	50 (16.9)	16 (15.8)	22 (22.5)
TE				
Grade A	235 (47.3)	155 (52.0)^a^	50 (49.5)^a^	30 (30.6)^b^
Grade B	128 (25.9)	65 (22.0)^a^	27 (26.7)^a,b^	36 (36.7)^b^
Grade C	133 (26.9)	77 (26.0)	24 (23.8)	32 (32.7)
Clinical pregnancies	234 (47.2)	136 (45.8)	47 (46.5)	51 (52.0)
Ongoing pregnancies	201 (40.5)	115 (38.7)	41 (40.6)	45 (45.9)
Follow-up data on live birth (ET date: April–December 2017)	246	149	53	44
Live birth	88 (35.8)	52 (34.9)	18 (34.0)	18 (40.9)
dBMov	3.84 ± 0.07 [0.16–25.54]	2.82 ± 0.05^a^ [0.16–8.49]	5.20 ± 0.13^b^ [0.734–14.57]	5.36 ± 0.17^b^ [0.63–25.54]
dBMov/(t3-t2)	0.366 ± 0.007 [0.014–1.000]	0.253 ± 0.004^a^ [0.014–0.727]	0.497 ± 0.015^b^ [0.071–1.000]	0.523 ± 0.016^b^ [0.061–1.000]

Categorical data are presented as n (%), continuous data are presented as mean ± standard error of the mean [range]. Different superscript letters (a, b) indicate a significant difference at *P* <  0.05. Abbreviations: dBMov, blastomere movement duration; dBMov/(t3-t2), ratio of the duration of blastomere movement at the 2-cell stage; ICM, inner cell mas; ET, embryo transfer; TE, trophectoderm

Embryos categorized with bouncing movement exhibited significantly higher developmental rates to the 8-cell, morula, blastocyst, and expanded-blastocyst stages than embryos with twist-and-crumble movement, especially from the 4-cell to the morula stage (Table 2 and Additional file 1). To adjust for potential statistical confounding bias, a multivariate logistic regression analysis was also performed. Multivariate logistic regression analysis also indicated low developmental rates in the twist-and-crumble group (Table 3). In contrast, the developmental rates were comparable between embryos with bouncing and wobbling movements. Furthermore, the morphological quality of the trophectoderm (TE) of embryos with twist-and-crumble movement was significantly lower than that of embryos with bouncing and wobbling movements, although the quality of the inner cell mass (ICM) was comparable among all groups (Table 2). The CPR, OPR, and LBR after SVBT were comparable among groups (Table 2). Multivariate logistic regression analysis also showed that movement type was not associated with OPR or LBR (Table 3).

**Table 3:** Adjusted odds ratio of blastomere movement type for embryonic development and pregnancy outcomes

Outcome	Movement type	Adjusted odds ratio (95% CI)	*P* value
Expanded blastocyst^a^	Bouncing	Reference	–
	Wobbling	0.763 (0.526–1.112)	0.1584
	Twist-and-crumble	0.476 (0.339–0.668)	< 0.0001
Ongoing pregnancy^b^	Bouncing	Reference	–
	Wobbling	1.182 (0.701–1.993)	0.5316
	Twist-and-crumble	1.514 (0.799–2.342)	0.1505
Live birth^b^	Bouncing	Reference	–
	Wobbling	1.303 (0.598–2.838)	0.5048
	Twist-and-crumble	1.295 (0.566–2.967)	0.5405

Notes: ^a^Confounding factors: female age, male age, and body mass index. ^b^Confounding factors: female age, male age, body mass index, number of previous embryo transfer cycles, and blastocyst morphological grade. Abbreviations: *CI* confidence interval

Multivariate logistic regression analysis revealed that an increased value of dBMov/(t3-t2) was significantly correlated with decreased developmental rates to the 8-cell, morula, blastocyst, and expanded blastocyst stages, especially from the 4-cell to the morula stage (Table 4 and Additional file 1). No correlation was observed between the value of dBMov/(t3-t2) and morphological quality of ICM (Grade A, 0.344 ± 0.011; B, 0.343 ± 0.014; C, 0.375 ± 0.021; *P* = 0.3065) or TE (Grade A, 0.335 ± 0.011; B, 0.359 ± 0.016; C, 0.363 ± 0.016; *P* = 0.0.2677). The CPR, OPR, and LBR were not correlated with the value of dBMov/(t3-t2) (Table 4).

**Table 4:** Adjusted odds ratio of the value of dBMov/(t3-t2) for embryonic development and pregnancy outcomes

Outcomes	Adjusted odds ratio (95% CI)	*P* value
Four-cell stage embryos^a^	0.131 (0.008–2.294)	0.1951
Eight-cell stage embryos^a^	0.265 (0.095–0.784)	0.0132
Morula^a^	0.274 (0.123–0.624)	0.0017
Blastocysts^a^	0.345 (0.176–0.678)	0.0019
Blastocysts expanded^a^	0.415 (0.217–0.794)	0.0077
Clinical pregnancy^b^	1.697 (0.659–4.368)	0.2711
Ongoing pregnancy^b^	1.578 (0.398–5.554)	0.5357
Live birth^b^	1.543 (0.321–6.946)	0.5773

Notes: ^a^Confounders: female age, male age, and body mass index. ^b^Confounders: female age, male age, body mass index, number of previous embryo transfer cycles, and blastocyst morphological grade. Abbreviations: *CI* confidence interval

Embryos were stratified into four groups according to the value of dBMov/(t3-t2): group A, dBMov/(t3-t2) < 0.217; group B, 0.217 ≤ dBMov/(t3-t2) < 0.300; group C, 0.300 ≤ dBMov/(t3-t2) < 0.420; and group D, 0.420 ≤ dBMov/(t3-t2) (Table 5). The cut-off values for group designation were determined using statistical software. The developmental rates to expanded blastocyst stage decreased significantly from group A to group D, and the Cochran-Armitage test confirmed a significant trend of declining expanded blastocyst rate with increasing dBMov/(t3-t2) value (*P* < 0.0001). On the other hand, the CPR, OPR and LBR were not correlated with the dBMov/(t3-t2) value.

**Table 5:** Embryonic and pregnancy outcomes after single vitrified-warmed blastocyst transfer, stratified by the value of dBMov/(t3-t2)

	No. of embryos	Female age	Male age	Blastocysts	Expanded blastocysts	No. of blastocysts transferred	Clinical pregnancy	Ongoing pregnancy	Follow-up data on live birth	Live birth
Group A [dBMov/(t3-t2) < 0.217]	208	39.1 ± 0.2	41.0 ± 0.4	170 (81.7)^a^	165 (79.3)^a^	121	58 (47.9)	48 (39.7)	72	27 (37.5)
Group B [0.217 ≤ dBMov/(t3-t2) < 0.300]	243	39.2 ± 0.2	41.2 ± 0.3	190 (78.2)^a^	178 (73.3)^a, b^	125	62 (49.6)	56 (44.8)	62	21 (33.9)
Group C [0.300 ≤ dBMov/(t3-t2) < 0.420]	232	38.9 ± 0.2	40.6 ± 0.3	178 (76.7)^a^	160 (69.0)^b, c^	118	51 (43.2)	42 (35.6)	55	19 (34.6)
Group C [0.420 ≤ dBMov/(t3-t2)]	283	39.1 ± 0.2	41.4 ± 0.4	193 (68.2)^b^	178 (62.9)^c^	132	63 (47.7)	55 (41.7)	57	21 (36.8)

Categorical data are presented as n (%), continuous data are presented as mean ± standard error of the mean. Abbreviations: dBMov/(t3-t2), duration of blastomere movement during the 2-cell stage. Different superscript letters indicate a significant difference at *P* < 0.05

## Discussion

This study demonstrates that embryos, those with twist-and-crumble movement, have a lower developmental rate after the 4-cell stage and poor morphological quality of the trophectoderm at the blastocyst stage. The poor trophectoderm morphology of twist-and-crumble embryos may influence the cumulative pregnancy rate, although the present study revealed no statistical correlation between the twist-and-crumble movement and pregnancy outcome after SVBT. Blastomere polarization in preimplantation embryos plays an important role in the patterning of embryos and proper embryonic development. In humans, disrupted polarization is associated with significantly reduced blastocyst formation and implantation rates [13]. We observed that the twist-and-crumble movement occurred concurrently with blastomere rolling. Although it is not known whether the twist-and-crumble movement interferes with or corrects cell polarity, our results suggest a potential association between improper blastomere polarization and decreased development in embryos with twist-and-crumble movement.

We have previously demonstrated that the prolongation of BMov is negatively associated with pregnancy outcomes following cleavage-stage embryo transfer on day 2; this extended BMov was associated with the delay of pronuclear fading and first cell division [10]. The present study also showed significant correlations between prolonged BMov and decreased blastocyst formation rates. The cytoskeleton plays a key role in organelle transport, segregation of chromosomes, cell division, motility, and signaling, which are crucial steps in cell cycle progression [14]. The distribution of cytoskeletal components is markedly changed in the cytoplasm of zygotes during the postfertilization period, and cytoskeletal reorganization after the first cell division is related to the success of embryonic development [15]. Therefore, prolongation of blastomere movement after the first cell division can be caused by abnormal cytoplasmic activity with respect to cytoskeleton modification and reorganization, which negatively affects subsequent blastocyst development. The LBRs following SVBT were not affected by BMov type or duration in the present study, suggesting that BMov is not correlated with pregnancy outcomes once transferable expanded blastocysts are produced and transferred. However, the correlation between blastocyst development and BMov duration rationalizes our previous finding of prolonged BMov being negatively correlated with pregnancy outcomes after cleavage-stage embryo transfer [10].

## Conclusions

The present study provides evidence that BMov after the first cell division significantly predicts blastocyst formation. Therefore, taken together with our previous findings, we suggest that analysis of BMov is effective for predicting pregnancy outcome after cleaved-embryo transfer on days 2 and 3, but not after blastocyst transfer on day 5. However, this study is limited by its retrospective nature; thus, further randomized controlled trial studies are required to validate our findings.

## Additional file

Additional file 1: Blastomere movement and embryonic development in each stage. (DOCX 21 kb)

## Abbreviations

BMI: Body mass index
BMov: Blastomere movement
CPR: Clinical pregnancy rate
dBMov: The duration of blastomere movement
dBMov/(t3-t2): Ratio of the duration of blastomere movement at the 2-cell stage
ET: Embryo transfer
ICM: Inner cell mass
ICSI: Intracytoplasmic sperm injection
LBR: Live birth rate
OPR: Ongoing pregnancy rate
SVBT: Single vitrified-warmed blastocyst transfer
TE: Trophectoderm

## References

[1] Meseguer M, Rubio I, Cruz M, Basile N, Marcos J, Requena A (2012). Embryo incubation and selection in a time-lapse monitoring system improves pregnancy outcome compared with a standard incubator: a retrospective cohort study. Fertil Steril.

[2] Kirkegaard K, Ahlstrom A, Ingerslev HJ, Hardarson T (2015). Choosing the best embryo by time lapse versus standard morphology. Fertil Steril.

[3] Rubio I, Galan A, Larreategui Z, Ayerdi F, Bellver J, Herrero J (2014). Clinical validation of embryo culture and selection by morphokinetic analysis: a randomized, controlled trial of the EmbryoScope. Fertil Steril.

[4] VerMilyea MD, Tan L, Anthony JT, Conaghan J, Ivani K, Gvakharia M (2014). Computer-automated time-lapse analysis results correlate with embryo implantation and clinical pregnancy: a blinded, multi-Centre study. Reprod BioMed Online.

[5] Meseguer M, Herrero J, Tejera A, Hilligsoe KM, Ramsing NB, Remohi J (2011). The use of morphokinetics as a predictor of embryo implantation. Hum Reprod.

[6] Kaser DJ, Racowsky C (2014). Clinical outcomes following selection of human preimplantation embryos with time-lapse monitoring: a systematic review. Hum Reprod Update.

[7] Barberet J, Chammas J, Bruno C, Valot E, Vuillemin C, Jonval L (2018). Randomized controlled trial comparing embryo culture in two incubator systems: G185 K-system versus EmbryoScope. Fertil Steril.

[8] Yang ST, Shi JX, Gong F, Zhang SP, Lu CF, Tan K (2015). Cleavage pattern predicts developmental potential of day 3 human embryos produced by IVF. Reprod BioMed Online.

[9] Ebner T, Hoggerl A, Oppelt P, Radler E, Enzelsberger SH, Mayer RB (2017). Time-lapse imaging provides further evidence that planar arrangement of blastomeres is highly abnormal. Arch Gynecol Obstet.

[10] Ezoe K, Ohata K, Morita H, Ueno S, Miki T, Okimura T, et al. Prolonged blastomere movement induced by the delay of pronuclear fading and first cell division adversely affects pregnancy outcomes after fresh embryo transfer on day 2: a time-lapse study. Reprod BioMed Online. 2019; In press. 10.1016/j.rbmo.2018.12.014 30853350

[11] Coughlan C, Ledger W, Wang Q, Liu F, Demirol A, Gurgan T (2014). Recurrent implantation failure: definition and management. Reprod BioMed Online.

[12] Kato K, Ezoe K, Yabuuchi A, Fukuda J, Kuroda T, Ueno S (2018). Comparison of pregnancy outcomes following fresh and electively frozen single blastocyst transfer in natural cycle and clomiphene-stimulated IVF cycles. Hum Reprod Open.

[13] Ajduk A, Zernicka-Goetz M (2016). Polarity and cell division orientation in the cleavage embryo: from worm to human. Mol Hum Reprod.

[14] Prosser SL, Pelletier L (2017). Mitotic spindle assembly in animal cells: a fine balancing act. Nat Rev Mol Cell Biol.

[15] Rivera RM, Kelley KL, Erdos GW, Hansen PJ (2004). Reorganization of microfilaments and microtubules by thermal stress in two-cell bovine embryos. Biol Reprod.

