# Supplementary material for: Blastomere movement post first cell division correlates with embryonic compaction and subsequent blastocyst formation
Source: Reprod Biol Endocrinol. 2019 May 15;17:44. doi: 10.1186/s12958-019-0488-5 (PMC6521525; doi:10.1186/s12958-019-0488-5)
Supplement: Supplementary file 1 — Blastomere movement and embryonic development in each stage. (DOCX 21 kb) [file 12958_2019_488_MOESM1_ESM.docx]

**Additional file 1. Blastomere movement and embryonic development in each stage.**

| Embryonic development | Type of blastomere movement | | |  | Duration of blastomere movement (dBMov ratio) | |
| --- | --- | --- | --- | --- | --- | --- |
|  | Bouncing | Wobbling | Twist-and-crumble |  | Adjusted odds ratio (95% CI)* | *P* value |
| From 4-cell to 8-cell stage | 516/543 (95.0)^a^ | 181/194 (93.3)^a, b^ | 202/227 (89.0)^b^ |  | 0.252 (0.090–0.752) | 0.0107 |
| From 8-cell to morula stage | 493/516 (95.5)^a^ | 166/181 (91.7)^a, b^ | 180/202 (89.1)^b^ |  | 0.240 (0.080–0.767) | 0.0128 |
| From morula to blastocyst stage | 420/493 (85.2) | 138/166 (83.1) | 150/180 (83.3) |  | 0.818 (0.333–2.099) | 0.6676 |
| From blastocyst to expanded blastocyst stage | 394/420 (93.8) | 131/138 (94.3) | 137/150 (91.3) |  | 0.912 (0.231–4.240) | 0.9010 |

Notes: *Confounders: female age, male age, and body mass index. Abbreviations: CI, confidence interval; dBMov, blastomere movement duration; dBMov/(t3-t2), ratio of the duration of blastomere movement at the 2-cell stage. Different superscript letters indicate a significant difference at *P* < 0.05.
